# 2229. Impact of Elexacaftor/tezacaftor/ivacaftor on Utilization of Antibiotics at a Single Pediatric Cystic Fibrosis Center

**DOI:** 10.1093/ofid/ofad500.1851

**Published:** 2023-11-27

**Authors:** Elizabeth C Elson, Brian R Lee, Alaina N Burns, Joshua C Herigon, Ann Wirtz, Stephanie Duehlmeyer

**Affiliations:** Children's Mercy Kansas City, Kansas City, Missouri; Children's Mercy Kansas City, Kansas City, Missouri; Children's Mercy Kansas City, Kansas City, Missouri; Children's Mercy Kansas City, Kansas City, Missouri; Children's Mercy Kansas City, Kansas City, Missouri; Children's Mercy Kansas City, Kansas City, Missouri

## Abstract

**Background:**

Intravenous (IV) and oral (PO) antibiotics (ABX) are standard treatments for pulmonary exacerbations (PEx) in people with cystic fibrosis (pwCF). Frequent ABX use may lead to adverse events and ABX resistance. Cystic fibrosis transmembrane conductance regulator modulator treatments have significantly reduced PEx in qualifying pwCF, but impact on ABX use is unknown. The approval of elexacaftor/tezacaftor/ivacaftor (ETI) in 2019 makes these treatments accessible to the majority of pwCF. The primary objective was to examine utilization of ABX pre-and post-ETI in pwCF managed at Children’s Mercy Kansas City’s (CMKC) CF center.

**Methods:**

A retrospective analysis of PO and IV ABX for CF PEx was performed at CMKC. The pre-ETI treatment period was defined as 1/1/2017-12/31/2019 and post-ETI treatment period as 1/1/2020-12/31/2022. A paired analysis including pwCF managed by CMKC during both periods was used to compare ABX courses pre- and post-ETI.

**Results:**

A total of 293 pwCF and 110 pwCF received PO and IV ABX during the study period, respectively. Of these, 224 pwCF managed by CMKC during both periods were included. IV ABX were received by 25 pwCF in both periods whereas 138 pwCF did not receive IV ABX in either period. There were 43 pwCF who received IV ABX only in the pre-ETI period compared to 18 pwCF who received IV ABX only in the post-ETI period (p=0.0021) resulting in a discordance of 27.2%. PO ABX were prescribed for 119 pwCF in both periods whereas 35 pwCF did not have PO ABX prescribed in either period. There were 52 pwCF who received PO ABXs in the pre-ETI period only compared to 18 pwCF who received PO ABX in the post-ETI period only (p=0.0001) resulting in a discordance of 31.3%. The number of patients receiving ABX directed against *Pseudomonas aeruginosa* and methicillin-resistant *Staphylococcus aureus* significantly decreased between periods. The median duration of ABX did not significantly change for IV (9.2 days vs. 8.9 days, p=0.958) or PO (14 days vs. 14 days, p=0.384) ABX during both periods.

**Conclusion:**

CMKC pwCF experienced a dramatic decline in prescribing both PO and IV ABX following approval of ETI. Future investigations are necessary to determine this trend nationwide and ascertain the overall impact from decreased ABX exposure in pwCF.

**Disclosures:**

**All Authors**: No reported disclosures

